# *PENS* approach for breaking bad news in the oncology outpatient setting: a real-world report

**DOI:** 10.1007/s00520-022-07458-9

**Published:** 2022-12-14

**Authors:** Sharada Mailankody, Prathika Sherigar, Ananth Pai, Ramnath Shenoy, Karthik Udupa, Shirley Lewis, Seema R. Rao

**Affiliations:** 1 Department of Medical Oncology, Kasturba Medical College, Manipal, Manipal Academy of Higher Education, Manipal, Karnataka India; 2 Department of Medical Oncology, Shirdi Sai Cancer Hospital, Kasturba Medical College, Madhavnagar, Manipal, 576104 Karnataka India; 3 Department of Radiotherapy and Oncology, Kasturba Medical College, Manipal, Manipal Academy of Higher Education, Manipal, Karnataka India; 4 Department of Palliative Medicine and Supportive Care, Kasturba Medical College, Manipal, Manipal Academy of Higher Education, Manipal, Karnataka India; 5 Bangalore Hospice Trust, Karunashraya, Bengaluru India

**Keywords:** Breaking bad news, Outpatient department, Low- and middle-income countries, Oncologists, Real world

## Abstract

**Purpose:**

Breaking bad news (BBN) is a vital part of oncology practice. We conducted this study to assess an abbreviated PENS protocol [**P**atient preference, **E**xplanation, **N**ext appointment, and **S**upport] for BBN in oncology outpatient (OP) settings.

**Methods:**

This observational study was conducted in a university teaching hospital, including cancer patients who were unaware of their condition and willing to discuss their disease status. The duration of BBN was the primary outcome. After the BBN session, patients filled a validated questionnaire; response scores of ≤ 13 were classified as content with BBN.

**Results:**

Fifty patients (mean age 53.7 years, range 28–76) were included in the study. The average duration of BBN was 6.1 (range 2–11) min. Assessed by the response score sum, 43 (86%) patients were satisfied with BBN. Only three (6%) of the discontented patients felt that the BBN duration was too short. Most (94%) of patients reported that they understood the information imparted during the BBN session. After the session, 36 (72%) patients admitted to either feeling the same or reassured compared to before the session. The oncologists also were comfortable with PENS.

**Conclusions:**

The PENS approach is a practical method for BBN, especially when the oncologists have higher OP workloads. More extensive trials are required to validate the protocol in other settings.

**Trial registration:**

Clinical Trial Registry of India (CTRI/2021/07/034707).

**Supplementary information:**

The online version contains supplementary material available at 10.1007/s00520-022-07458-9.

## Introduction

India has seen an increasing incidence of cancer over the last decades, resulting in a definite shortage of trained oncologists [1–3]. Oncology services are part of tertiary care, accentuating the deficiencies in rural areas. In addition, oncologists in low- and middle-income countries (LMIC) like India have higher workloads than high-income countries (HIC) [1, 3]. The time available for each patient may be limited in such practice settings. Breaking bad news (BBN) is a vital part of oncology practice and is time-consuming. Indian oncologists usually have no formal training for challenging communications and learn to break bad news on the job. The BBN session profoundly impacts patient experience and trust in the oncologist and can do lasting emotional damage if undertaken carelessly [4].

Many family members may be involved in the decision-making process for oncology treatments and accompany the patient to the oncology clinic for consults. The Indian family system is collectivistic, with primarily joint families, compared to the individualistic approach from the West [5]. Cancer patients differ in their outlook and preference regarding receiving information about their illness, requiring distinct communication models in different parts of the world [6–12]. The ethical principles of medicine and patient autonomy require the patient to know the diagnosis and play a role in the decision-making [13]. Though most patients in India seem to want information about their disease conditions, family members are sometimes determined to withhold the same, leading to collusion [14–16]. All the above factors make patients anxious and emotional when coming for outpatient (OP) consults, further exacerbated by the long travel, waiting period to meet the oncologists, and anxiety regarding the diagnosis.

One of the widely used protocols for BBN in the oncology setting is the SPIKES approach, consisting of the correct **S**etting, assessing the **P**erception of the patient, **I**nvitation from the patient to break the news, imparting **K**nowledge, empathy for their **E**motions, and **S**trategy/summary of the session [17]. BREAKS is another protocol, an acronym for **B**ackground, **R**apport, **E**xplore, **A**nnounce, **K**indle, and **S**ummarize [18]. Another protocol, ABCDE, consists of **A**dvance preparation, **B**uilding a therapeutic relationship, good **C**ommunication, **D**ealing with reactions, and **E**ncouraging emotions [19]. These protocols are lengthy and involve multiple steps. The physicians may be able to choose a good setting and time for BBN for inpatients (IP). However, in busy oncology OP departments, it may not be feasible for an oncologist to spend a very long time with each patient. The doctor may be unable to arrange the ideal setting for each patient for BBN either. There is an unmet need for a validated BBN protocol suitable for the situations mentioned above.

Considering the limited time and the available resources with the oncologists in the busy OP setting, we proposed an abbreviated protocol, PENS, for BBN in the OP setting [19]. The PENS approach is based on the basic tenet of patient autonomy since the first step is to elicit **P**atient **p**reference regarding the BBN session. The rest of the steps are simple and include an **E**xplanation of the disease condition, scheduling the **N**ext appointment, and offering **S**upport to the patient. The PENS approach suggests delivering only the basic necessary information in the first visit, helping the patients assimilate information better and make important decisions regarding the initial treatment. Thus, the entire “informing” session may be divided into several sittings. As the patient becomes more comfortable with the oncologist with repeated discussions, patients/relatives can come up with further relevant queries. Though this approach may need a second visit to the oncologist within a short time for further discussion, most of the patients require frequent OP visits before treatment initiation, for workup completion.

In the OP setting BBN session, there needs to be a delicate balance between informing the patient regarding the diagnosis and treatment plan and not antagonizing the immediate family members in their desire to protect the patients [20–22]. Our training modules focus very little on communication enhancement [22–24]. All oncology centers may not have access to trained psycho-oncology or palliative care physicians who undergo formal training in difficult communication situations [24]. Our study reports the use of the PENS approach in the OP setting and explores patient and physician satisfaction with the PENS approach.

## Materials and methods

This study was a single-center observational study, in a university teaching hospital, Kasturba Medical College, Manipal, to evaluate an abbreviated protocol for breaking bad news in cancer patients. The detailed PENS protocol has been published already [19]. The trial investigator circulated the paper on PENS protocol to the participating oncologists. Further, the investigator demonstrated the PENS protocol to the participating oncologists. Thus, the oncologists involved in the study were trained in the PENS protocol [19]. The step of offering support included a referral to the department of palliative medicine and supportive care, as and when required, for expert psychosocial support. All new patients presenting to the medical oncology OP between July 1, 2021, and November 15, 2021, were screened for inclusion in the study. Those patients who were unaware of their diagnosis but were willing to discuss their disease and treatment plans and those who had to be informed about their disease progression were included in the study. To include only eligible patients, the participating oncologists asked the patients whether they knew about their condition and disease status. Further, oncologists also enquired regarding their willingness to discuss the diagnosis and treatment options, ensuring that only suitable patients were included in the study. The accompanying persons/relatives were also automatically a part of the BBN discussion unless the patient specifically requested their exclusion from the session. Written informed consent was obtained from all study participants after the session of BBN before the study questionnaire was filled. Patients who knew their diagnosis and prognosis or unwilling to be part of the BBN session and pediatric and pregnant patients were excluded from the study.

The participating oncologists used the PENS approach for BBN during the study period for all patients satisfying the inclusion criteria. The oncologists noted and documented the time at the start and end of the BBN session. The BBN duration recorded did not include the time taken for history, clinical examination, and review of reports. In many cases, at the request of the family, the financial aspects of treatment were discussed in a separate session and not included in the BBN session. The amount of information imparted during the BBN session was based on the oncologists’ discretion and was individualized for patients.

As there is no validated protocol for assessing patient satisfaction after a BBN session, the authors designed a suitable questionnaire. Experts in the field, including palliative care physicians and experienced medical oncologists, validated the questionnaire content. After completing the first appointment with the oncologist, the patients participating in the study filled out the validated six-item questionnaire to assess the acceptability of the PENS protocol (Annexure). The questionnaire consisted of 6 questions with answers based on Likert method. The responses were scored, with higher scores indicating lesser satisfaction. The maximum score indicating favorable response was 13; a score ≤ 13 was hence taken as a measure of satisfaction. At the end of the study, all the participating oncologists also filled out a four-item questionnaire on their comfort level and satisfaction with the PENS approach for BBN (Annexure).

### Statistical analysis

Since this study was planned to assess the use of the new approach, we evaluated 50 patients presenting to the oncology OP requiring BBN. Descriptive statistics were used to summarize the data frequency for baseline characteristics. The Chi-square test was used to test the correlation between patient satisfaction and age, Eastern Cooperative Oncology Group Performance Status (ECOG PS), the accompanying bystander, home district, and education status. A *p* value of < 0.05 was considered significant.

### Ethical considerations

The study protocol was approved by the Institutional Ethics Committee (IEC) of Kasturba Medical College and Kasturba Hospital (IEC 279/2021) and was registered with the Clinical Trial Registry of India (CTRI/2021/07/034707). It was conducted according to the Declaration of Helsinki and good clinical practice guidelines.

## Results

**Fig. 1:**
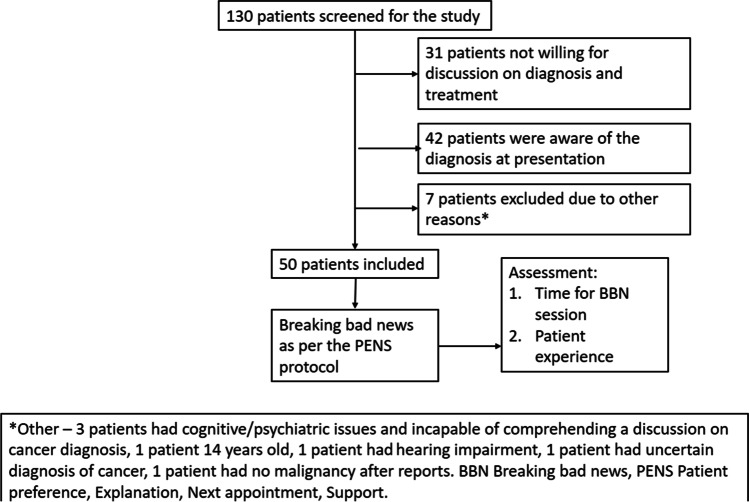
Study methodology

Of the 130 screened patients, 50 patients were included in the study (Fig. 1). Of the 80 excluded patients, 31 (38.8%) were unwilling to discuss further care. Forty-two (52.5%) patients were aware of the diagnosis before coming to the OPD.

The mean age of the patients was 53.7 (range 28–76) years. The Eastern Cooperative Oncology Group (ECOG) performance status was one in 37 (74%). Forty-three (86%) patients visited the medical oncology department for the first time. The majority of the patients (39, 78%) had only less than a high school education. Among the 28 already- staged patients, 16 (57%) had stage IV malignancy. Except for one patient, close relatives (offspring/sibling/spouse/other close relatives) accompanied the patient to the oncology appointment. The bad news in 90% (45) patients was a newly diagnosed malignancy.

The baseline characteristics are detailed in Table 1.

**Table 1:** Baseline characteristics of included patients

Baseline characteristics	No of patients (%)
Age	
≤ 60 years	37 (74)
> 60 years	13 (26)
ECOG PS	
0	6 (12)
1	37 (74)
2	5 (10)
3	2 (4)
4	0 (0)
Male/female	
Women	22 (44)
Men	28 (56)
Education*	
Matriculation or less	39 (78)
Post matriculation	8 (16)
Postgraduate or professional	3 (6)
Accompanying attender	
Spouse	12 (24)
Offspring	23 (46)
Other close relatives (son-in law, nephew/uncle, grandson)	14 (28)
Friend	1 (2)
Patients home district	
Neighboring districts	16 (32)
> 60 km away	34 (68)
Setting**	
OP	38 (76)
IP	12 (24)
OP visit type	
New patients/first visit	43 (86)
Patients with prior visits	7 (14)
Type of BBN	
Newly diagnosed	45 (90)
Progressive disease	5 (10)
Stage of disease	
1	2 (4)
2	4 (8)
3	6 (12)
4	16 (32)
Awaited/NA	22 (44)
Type of malignancy	
Solid tumor	44 (88)
Hematological malignancy	6 (12)

*Matriculation standard implies at least 10 years of formal education, until 15–16 years of age

**The patient is seen on an OP basis in the oncology department, admitted under some other department

*ECOG PS*, Eastern Cooperative Oncology Group Performance Status; *std*, standard; *km*, kilometers; *OP*, outpatient; *IP*, inpatient; *BBN*, breaking bad news

### Duration of the BBN session

The average time taken for the BBN session was 6.1 (range 2–11) min.

### Patient satisfaction

Forty-three (86%) patients were satisfied with the BBN session, as assessed by the sum of the response scores. Of the seven (14%) patients who were unhappy with the session, only three (6%) of the patients felt that the interview was too short. Exploring the individual aspects of the BBN session, most (94%) of the patients reported that they were satisfied with the BBN session overall. Forty-nine (98%) patients claimed to have understood most of the conversation or at least half of the communication. Only three patients reported having many doubts after the BBN session; the rest were clear about the information conveyed to them or were confident about the doctor addressing their concerns in the next visit. All the patients felt that the doctor was approachable, interested, and willing to listen to their problems. Regarding the time taken for the interview, 10 (20%) of the patients felt too little time was taken. After the session, 36 (72%) patients admitted to feeling the same or less anxious or reassured and confident when compared to before the session.

### Relation of patient satisfaction with other characteristics

We assessed the relationship between factors like age (> 60 years), home district, accompanying person, ECOG PS, and the education with satisfaction status. The education level of the patient was classified as less than matriculation (at least 10 years of formal education) or higher. The education level of the patient was inversely correlated with patient satisfaction. There was no significant correlation with other factors (Table 2).

**Table 2:** Correlation of patient satisfaction with other characteristics

	Patient satisfied	Unsatisfied	Chi-square	*p* value
Age				
> 60 years	10	3	1.2	0.27
< 60 years	33	4		
Accompanying attender				
Close relative	35	5	0.4	0.54
Others	8	2		
ECOG PS**				
Good (0–1)	36	7	0.1	0.79
Poor	7	0		
Patient home district***				
Neighboring district	15	1	1.2	0.28
> 60 km away	28	6		
Education****				
Less than matriculation	36	3	5.9	0.02
Higher education	7	4		

*ECOG PS*, Eastern Cooperative Oncology Group Performance Status

*Close relative was either spouse, offspring, or a sibling

**ECOG PS good 0–1, poor > 2

***Whether the home district of the patient was in either the same district or a neighboring district in relation to the study center

****Matriculation standard implies at least 10 years of formal education, until the age of 15–16 years. Patient education correlated significantly with the patient satisfaction (*p*<0.05)

### Doctor acceptance

All the four medical oncologists [mean age 37.5 (range 35–40)] in the department were included in the study. Of the doctors involved in the study, prior experience in the oncology field was 5–13 years (mean nine years). All the oncologists involved were very comfortable [3 (75%)] or somewhat comfortable [1 (25%)] using this new approach to breaking bad news and were very satisfied [4 (100%)] with the BBN session. The oncologists also felt “very confident” [2(50%)] or “somewhat confident” [2 (50%)] about identifying the patient emotions during the BBN sessions.

## Discussion

Due to a higher workload, oncologists in India face distinct challenges in doctor-patient communications compared to HIC [25]. There is an unmet need for an abbreviated protocol tailored for the Indian setting to break bad news in the busy oncology OP [19]. Our study explored the patient-centered, culturally appropriate PENS approach for breaking bad news to patients. Our results show that the first BBN session takes around 6 min, though the interview continued beyond 6 min to explore other concerns. The duration seems appropriate in the context of an average oncology OP patient load. There is also reasonable patient satisfaction (86%) with the novel protocol for BBN. The patient satisfaction with the BBN session seems related to patients’ educational level. Our study is the first study in India to assess an indigenous approach to breaking bad news in our OP settings.

The time taken is a critical issue in busy oncology practice, where each oncologist has to see around 20–50 patients per day, as in an LMIC [1, 25]. In this study, we report the real-world outcomes of the duration of the initial diagnosis/progression disclosure session. The time spent with each patient depends on factors like age and educational status, disease-related factors, appointment setting, accompanying relatives, and the amount of imparted details. Visit length has been linked to many factors, including treatment adherence, which may affect patient outcomes [26, 27]. Other studies have reported varying durations of 22–37 min for BBN or first OP visits, albeit in different settings than ours [28, 29]. The current study reported only the BBN session interval; nevertheless, there are no Indian studies on oncology OP BBN session durations. Our study is a single-center study from a rural healthcare facility, with most patients having only primary education, which may account for the shorter BBN durations. Breaking the BBN session into multiple smaller sessions may be particularly appropriate for such patients. For an average Indian family, finances may be the primary determinant of treatment, as studies have revealed extremely high out-of-pocket and catastrophic expenditures for cancer care in India [30, 31]. Hence, when the family insists, the oncologist has to speak about some aspects of treatment like finances, without including the patient in the conversation, which might have also contributed to the shorter session times. In 44% of the patients included in the study, the disease stage was unknown. Hence, the extent of information imparted to each patient was also variable at the first visit, as is seen in real-world practice.

The essence of the PENS approach is eliciting patient preference at the beginning of the BBN session. Though the patient is the center of the cancer treatment, in Indian society with strong family ties, the family members often request the oncologist to withhold vital diagnostic information from the patient [14]. However, in the PENS approach, the patient can opt out of the BBN session, designating a responsible relative for further discussion. This step is in line with the ethical principles of patient autonomy, and oncologists can discuss the diagnosis freely with the authorized representative. In this study, of the 80 excluded patients, 31 (38.8%) patients were unwilling to join the BBN session, preferring not to know or discuss with their relatives later. Many studies on cancer patients, including Indian patients, have revealed that, in general, patients want information on the disease status and treatment plan [6–10, 32].

Patient satisfaction with the session was also assessed in the study, with 43 (86%) of the patients revealing reasonable satisfaction with the protocol. A sizeable proportion of the patients reported a good understanding of the BBN session. Though 20% of patients felt that the duration was too short, all patients felt that the doctor was interested in listening to their concerns. The above findings underline that the time is not the primary factor in deciding the effectiveness of the BBN session. If a patient understands the information provided and feels supported by the oncologist, the BBN session can then be considered adequate. The PENS approach emphasizes the discussion of practical aspects of cancer care, like access to a cancer care center, which is more of a concern in India than the actual treatment process or outcomes [19, 31, 33].

Though the session provided life-altering news, many patients (72%) did not report increased anxiety after the session; the session with the oncologists reassured them instead (60%). Half of the patients were confident that the oncologist would clarify further concerns in the subsequent visits, emphasizing that patients also understand the limitation that not all the information can be imparted in a single session in our setting. The education status of the patient was inversely related to satisfaction with the BBN session in the current study. Research shows that the patient educational status changes the priorities in a doctor-patient communication session, with educated patients preferring a more problem-oriented session than the emotional component [34]. There may also be a negative communication experience in patients with lower language skills [35]. In the current study, however, most (78%) patients had lower educational status. Our study assessed the actual patient experience after an oncology appointment in a real-world setting, which is significant in the era of patient-reported outcomes [36].

The protocol was acceptable for the oncologists, as per this study. Prior studies suggest a lacuna in doctors’ confidence in delivering bad news effectively [23]. Providing information on disease progression to treated patients is often emotionally draining to the oncologists, especially when communication is ineffective [16, 21]. In the long run, this workplace distress may lead to burnout of oncologists [23]. Each patient and situation is distinct, and BBN requires dynamic assessment of the problem and contextual adaptation of the available communication protocols [12]. A brief and pragmatic approach like PENS may be acceptable to most oncologists. A practical protocol for BBN, particularly the potentially challenging sessions, will help improve the patient-physician relationship and decrease distress [20, 37, 38]. Unpleasant BBN sessions may also add to oncologists’ work pressures and may be linked to workplace frustration and burnout [21, 39, 40].

A few approaches to improving communication in oncology settings suggested are the inclusion of communication skills modules in oncology training programs, using validated talking maps for communication regarding serious illnesses and patient-reported outcomes for communication assessment [17, 18, 36, 41, 42]. However, the pattern of care and the patient profile is heterogeneous in different countries and even within the same country. Hence, it is essential to adapt the protocols and develop locally suitable protocols for the common problems faced in daily clinical practice [11, 12, 21, 43, 44]. PENS, an abbreviated approach to BBN, is one such protocol specially designed for use in the Indian OP setting. Though PENS was devised for use in oncology, other specialties requiring abbreviated BBN protocols in OP situations may also find it pertinent. Different approaches for BBN like SPIKES, BREAKS, and ABCDE are five–six step protocols. Compared with the PENS approach, they are more comprehensive and based upon a detailed understanding and acknowledgement of patient emotions. In contrast, PENS emphasizes patient autonomy and imparting necessary information for immediate decision-making [19]. Moreover, arrangement for an “ideal setting” for BBN, as suggested by SPIKES, BREAKS, and ABCDE approaches, may not be feasible in a crowded oncology OP. Since oncologists are not formally trained in doctor-patient communication, PENS approach may also be easily used by newly trained or trainee oncologists [17]–[19, 45]. PENS seems more relevant for the distinct socioeconomic and OP clinical milieu in India. A locally relevant BBN protocol is an unmet need, which led to the proposal of a shorter approach [19].

The strength of this study is that it reports the protocol practicability in a real-world busy oncology OP. The study center is a part of a comprehensive cancer care center and is affiliated to the university teaching hospital. Like many oncology centers in India, there is predominant delivery of clinical services, along with research activities in our center. Different facets of patient satisfaction were evaluated objectively to decide the outcome of the BBN session, and the doctor’s satisfaction was also assessed. However, this was a small study with a heterogeneous sample, and the relation of BBN sessions with other factors could not be evaluated due to the small sample size. As there were no standard validated tools for assessment of patient satisfaction with BBN session, a questionnaire was used to assess the same. Experts validated the content of the questionnaire. The current study was a single-center study, and the protocol requires validation in a larger sample for more acceptability. Nevertheless, it is the first study exploring the approach for BBN in the Indian oncology OP [1].

There is an unmet need for validated communication protocols and skills training in India for difficult communication situations. Including the AETCOM (attitude, ethics, and communication) module in the undergraduate education curriculum in India is a significant step in this direction. There is a need for further studies on effective communication strategies in our settings and suitable for our patients. Future prospective validation studies of such local protocols with an evaluation of patient-reported outcomes for assessment of efficacy are required for bringing about widespread changes in the oncology community.

## Conclusions

The PENS approach is a practical and abbreviated method for breaking bad news in an oncology OP. The patients and the oncologists were satisfied with this abbreviated protocol, which can be easily adopted in busy OP settings.

## Supplementary information

Below is the link to the electronic supplementary material.

Supplementary file1 (DOCX 16 KB)

## Data Availability

The data is available with the first and the corresponding author and will be provided on request.

## Abbreviation

Abbreviation: Full form
BBN: Breaking bad news
OP: Outpatient
IEC: Institute Ethics Committee
IP: Inpatient
PRO: Patient-reported outcomes
AETCOM: Attitude ethics and communication
ECOG: Eastern Cooperative Oncology Group
LMIC: Low- and middle-income countries
HIC: High-income countries
