# Supplementary material for: PENS approach for breaking bad news in the oncology outpatient setting: a real-world report
Source: Support Care Cancer. 2022 Dec 14;31(1):13. doi: 10.1007/s00520-022-07458-9 (PMC9747823; doi:10.1007/s00520-022-07458-9)
Supplement: Supplementary file 1 — Supplementary file1 (DOCX 16 KB) [file 520_2022_7458_MOESM1_ESM.docx]

**PATIENT QUESTIONNAIRE**

A. How do you feel about the conversation with the doctor?

1. Very satisfied
2. Somewhat satisfied
3. Neither satisfied nor dissatisfied
4. Somewhat dissatisfied
5. Very dissatisfied

B. How would you rate your understanding of the disease and treatment after talking to the doctor?

1. Understood everything clearly
2. Understood most of it
3. Understood half of it.
4. Understood only a little
5. Did not understand anything

C. After talking to the doctor, what do you feel about the taking decisions about the treatment?

1. No doubts, things are very clear and I have all the information needed to take a decision.
2. A few doubts, I am sure doctor will speak more about them during the next visit
3. Many doubts.

D. Was the doctor interested and willing to listen to your concerns?

1. Very willing, was approachable
2. Mostly willing
3. Somewhat willing
4. Not at all willing

E. What did you feel about the time duration taken of the interview?

1. Too much time
2. A lot of time
3. Enough time
4. Too less time
5. No time at all

F. How do you feel after the session?

1. Feeling reassured and confident
2. Feeling less anxious
3. Same as before the interview
4. A little more anxious and distressed
5. More anxious and distressed

**DOCTOR QUESTIONNAIRE**

A. How long have you been practicing oncology?

____ years (including the oncology training)

B. How comfortable were you with the PENS protocol?

1. Very comfortable
2. Somewhat comfortable
3. Neither comfortable nor uncomfortable
4. Somewhat uncomfortable
5. Very uncomfortable

C. How do you feel about the BBN sessions?

1. Very satisfied
2. Somewhat satisfied
3. Neither satisfied nor dissatisfied
4. Somewhat dissatisfied
5. Very dissatisfied

D. How confident did you feel about identifying with patient emotions during the BBN session?

- 1. Very confident
  2. Somewhat confident
  3. Confident
  4. A little uncertain
  5. Very uncertain
